# 6-Butyl-5-(4-methyl­phen­oxy)-3-phenyl-3*H*-1,2,3-triazolo[4,5-*d*]pyrimidin-7(6*H*)-one

**DOI:** 10.1107/S160053681004300X

**Published:** 2010-10-31

**Authors:** Hong-Mei Wang, Shou-Heng Deng, Xiao-Hua Zeng, Ping Chen, Li-Li Chen

**Affiliations:** a Institute of Medicinal Chemistry, Hubei Medical University, Shiyan 442000, People’s Republic of China; b Center of Oncology, People’s Hospital affiliated with Hubei Medical University, Shiyan 442000, People’s Republic of China

## Abstract

In the title compound, C_21_H_21_N_5_O_2_, the triazolopyrimidine ring system is essentially planar [maximum displacement = 0.021 (4) Å] and forms dihedral angles of 41.17 (9) and 67.99 (8)° with the phenyl and benzene rings, respectively. The *n*-butyl side chains is disordered over two positions with an ccupancy ratio of 0.77:0.23. An intra­molecular C—H⋯O hydrogen-bonding inter­action stabilizes the mol­ecular conformation. In the crystal, mol­ecules are linked by inter­molecular C—H⋯O and C—H⋯N hydrogen bonds into a three-dimensional network. In addition, π–π stacking inter­actions involving the triazole and pyrimidine rings of adjacent mol­ecules are observed, with centroid–centroid distances of 3.545 (1) Å.

## Related literature

For the synthesis and biological activity of 8-aza­guanine derivatives, see: Roblin *et al.* (1945 ▶); Ding *et al.* (2004 ▶); Mitchell *et al.* (1950 ▶); Levine *et al.* (1963 ▶); Montgomery *et al.* (1962 ▶); Yamamoto *et al.* (1967 ▶); Bariana (1971 ▶); Holland *et al.* (1975 ▶); Zeng *et al.* (2010 ▶). For related structures, see: Ferguson *et al.* (1998 ▶); Li *et al.* (2004 ▶); Zhao, Xie *et al.* (2005 ▶); Zhao, Hu *et al.* (2005 ▶); Zhao, Wang & Ding (2005 ▶); Chen & Shi (2006 ▶); Maldonado *et al.* (2006 ▶); Xiao *et al.* (2007 ▶); Wang *et al.* (2006 ▶, 2008 ▶); Zeng *et al.* (2006 ▶, 2009 ▶).
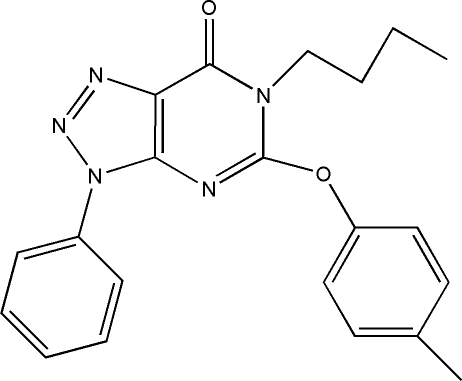


## Experimental

### 

#### Crystal data

C_21_H_21_N_5_O_2_
*M* *_r_* = 375.43
Monoclinic, 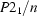

*a* = 11.0954 (10) Å
*b* = 16.4478 (15) Å
*c* = 11.3484 (11) Å
β = 107.643 (1)°
*V* = 1973.6 (3) Å^3^
*Z* = 4
Mo *K*α radiation
μ = 0.09 mm^−1^
*T* = 298 K
0.20 × 0.20 × 0.20 mm

#### Data collection

Bruker SMART CCD area-detector diffractometer
Absorption correction: multi-scan (*SADABS*; Sheldrick, 1996 ▶) *T*_min_ = 0.983, *T*_max_ = 0.985
20458 measured reflections
3876 independent reflections
2663 reflections with *I* > 2σ(*I*)
*R*_int_ = 0.055

#### Refinement

*R*[*F* ^2^ > 2σ(*F* ^2^)] = 0.054
*wR*(*F* ^2^) = 0.182
*S* = 1.08
3876 reflections
293 parameters
11 restraints
H-atom parameters constrained
Δρ_max_ = 0.56 e Å^−3^
Δρ_min_ = −0.24 e Å^−3^

### 

Data collection: *SMART* (Bruker, 2001 ▶); cell refinement: *SAINT* (Bruker, 2001 ▶); data reduction: *SAINT*; program(s) used to solve structure: *SHELXS97* (Sheldrick, 2008 ▶); program(s) used to refine structure: *SHELXL97* (Sheldrick, 2008 ▶); molecular graphics: *PLATON* (Spek, 2009 ▶); software used to prepare material for publication: *SHELXTL* (Sheldrick, 2008 ▶).

## Supplementary Material

Crystal structure: contains datablocks global, I. DOI: 10.1107/S160053681004300X/rz2502sup1.cif

Structure factors: contains datablocks I. DOI: 10.1107/S160053681004300X/rz2502Isup2.hkl

Additional supplementary materials:  crystallographic information; 3D view; checkCIF report

## Figures and Tables

**Table 1:** Hydrogen-bond geometry (Å, °)

*D*—H⋯*A*	*D*—H	H⋯*A*	*D*⋯*A*	*D*—H⋯*A*
C12—H12*A*⋯O2	0.97	2.50	3.048 (5)	116
C2—H2⋯O1^i^	0.93	2.53	3.230 (3)	133
C3—H3⋯N2^ii^	0.93	2.61	3.535 (2)	174

Symmetry codes: (i) 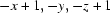; (ii) 
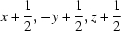
.

**Fig. 1.**
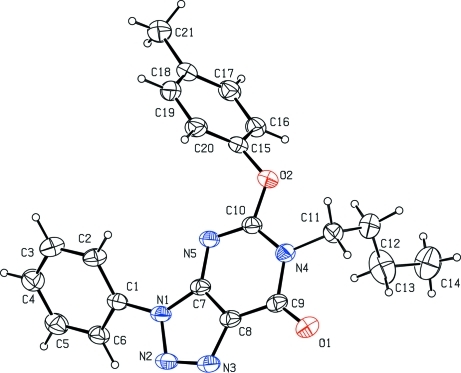
The molecular structure of the title compound showing the atom-labeling scheme. Displacement ellipsoids are drawn at 50% probability level. H-atoms are represented by circles of arbitrary size. Only the major component of the disorder is shown.

## supplementary crystallographic information

### Comment

The derivatives of heterocycles containing 8-azaguanine system, which are well known bioisosteres of guanine, are of great importance because of their remarkable biological properties. Some of these activities include antimicrobial or antifungal activities (Roblin *et al.*, 1945; Ding *et al.*, 2004; Zeng *et al.*, 2010), encephaloma cell inhibitor activity (Mitchell *et al.*, 1950; Levine *et al.*, 1963), antileukemic activity (Montgomery *et al.*, 1962), hypersusceptibility inhibitor activity and acesodyne activity (Yamamoto *et al.*, 1967; Bariana, 1971; Holland *et al.*, 1975). In recent years, Ding's group has been engaged in the preparation of derivatives of 8-azaguanine *via* aza-Wittig reaction of beta-ethoxycarbonyl iminophosphoranes with aromatic isocyanates (Zhao, Xie *et al.*, 2005). As a continuation of our research for new biologically active heterocycles, the title compound was obtained from beta-ethoxycarbonyl iminophosphorane with alphalic isocyanate, and structurally characterized in this context.

In the title compound (Fig. 1), bond lengths and angles within the triazolopyrimidinone moiety are in good agreement with those observed for closely related structures (Zhao, Hu *et al.*, 2005; Zhao, Wang & Ding, 2005). As reported for related compounds (Ferguson *et al.*, 1998; Li *et al.*, 2004; Maldonado *et al.*, 2006; Zeng *et al.*, 2006, 2009; Wang *et al.*, 2006, 2008; Xiao *et al.*, 2007; Chen & Shi, 2006), the triazolopyrimidine ring system is essentially planar, with a maximum displacement of 0.021 (4) Å for atom C8, and forms dihedral angles of 41.17 (9) and 67.99 (8)° with the C1–C6 and C15–C20 rings, respectively. There exists an intramolecular C—H···O hydrogen bonding interaction stabilizing the molecular conformation. In the crystal packing, molecules are linked by intermolecular C—H···O and C—H···N hydrogen bonds (Table 1). In addition, π–π stacking interactions involving the triazole and pyrimidine rings of adjacent molecules are observed, with cenroid-to-centroid distances of 3.545 (1) Å.

### Experimental

To the solution of carbodiimide in CH_2_Cl_2_/CH_3_CN (1:4 *v*/*v*, 15 ml) prepared according to the literature method (Zeng *et al.*, 2006), was added 4-methylphenol (3 mmol) and excess K_2_CO_3_, and the reaction mixture was stirred for 12 h. The solvent was removed under reduced pressure and the residue was recrystallized from EtOH to give the title compound (yield 93%; m.p. 406 K). Elemental analysis: calculated for C_21_H_21_N_5_O_2_: C, 67.18; H, 5.64; N, 18.65%. Found: C, 66.62; H, 5.98; N, 18.13%. Crystals suitable for single crystal X-ray diffraction analysis were obtained by slow evaporation of a hexane/dichloromethane (1:3 *v*/*v*) solution at room temperature.

### Refinement

H atoms were placed at calculated positions and treated as riding atoms, with C—H = 0.93–0.97 Å, and *U*_iso_(H) = 1.2*U*_eq_(C) for CH or 1.5*U*_eq_(C) for CH_3_. The *n*-butyl side chain is disordered over two positions with occupancy factors of 0.77:0.23. During the refinement, the adjacent and interval C—C distances involving the disordered carbon atoms were restrained to be 1.54 (1)Å and 2.45 (2) Å, respectively, by using the command *DFIX*.

### Crystal data

**Table:**

C_21_H_21_N_5_O_2_	*F*(000) = 792
*M**_r_* = 375.43	*D*_x_ = 1.263 Mg m^−^^3^
Monoclinic, *P*2_1_/*n*	Mo *K*α radiation, λ = 0.71073 Å
Hall symbol: -P 2yn	Cell parameters from 6350 reflections
*a* = 11.0954 (10) Å	θ = 2.3–25.8°
*b* = 16.4478 (15) Å	µ = 0.09 mm^−^^1^
*c* = 11.3484 (11) Å	*T* = 298 K
β = 107.643 (1)°	Block, colourless
*V* = 1973.6 (3) Å^3^	0.20 × 0.20 × 0.20 mm
*Z* = 4	

### Data collection

**Table:**

Bruker SMART CCD area-detector diffractometer	3876 independent reflections
Radiation source: fine-focus sealed tube	2663 reflections with *I* > 2σ(*I*)
graphite	*R*_int_ = 0.055
φ and ω scans	θ_max_ = 26.0°, θ_min_ = 2.3°
Absorption correction: multi-scan (*SADABS*; Sheldrick, 1996)	*h* = −13→13
*T*_min_ = 0.983, *T*_max_ = 0.985	*k* = −20→20
20458 measured reflections	*l* = −13→13

### Refinement

**Table:**

Refinement on *F*^2^	Primary atom site location: structure-invariant direct methods
Least-squares matrix: full	Secondary atom site location: difference Fourier map
*R*[*F*^2^ > 2σ(*F*^2^)] = 0.054	Hydrogen site location: inferred from neighbouring sites
*wR*(*F*^2^) = 0.182	H-atom parameters constrained
*S* = 1.08	*w* = 1/[σ^2^(*F*_o_^2^) + (0.1122*P*)^2^ + 0.0419*P*] where *P* = (*F*_o_^2^ + 2*F*_c_^2^)/3
3876 reflections	(Δ/σ)_max_ < 0.001
293 parameters	Δρ_max_ = 0.56 e Å^−^^3^
11 restraints	Δρ_min_ = −0.24 e Å^−^^3^

### Special details

**Table:**

Geometry. All e.s.d.'s (except the e.s.d. in the dihedral angle between two l.s. planes) are estimated using the full covariance matrix. The cell e.s.d.'s are taken into account individually in the estimation of e.s.d.'s in distances, angles and torsion angles; correlations between e.s.d.'s in cell parameters are only used when they are defined by crystal symmetry. An approximate (isotropic) treatment of cell e.s.d.'s is used for estimating e.s.d.'s involving l.s. planes.
Refinement. Refinement of *F*^2^ against ALL reflections. The weighted *R*-factor *wR* and goodness of fit *S* are based on *F*^2^, conventional *R*-factors *R* are based on *F*, with *F* set to zero for negative *F*^2^. The threshold expression of *F*^2^ > σ(*F*^2^) is used only for calculating *R*-factors(gt) *etc*. and is not relevant to the choice of reflections for refinement. *R*-factors based on *F*^2^ are statistically about twice as large as those based on *F*, and *R*- factors based on ALL data will be even larger.

### Fractional atomic coordinates and isotropic or equivalent isotropic displacement parameters (Å^2^)

**Table:**

	*x*	*y*	*z*	*U*_iso_*/*U*_eq_	Occ. (<1)
C1	0.42419 (17)	0.24051 (13)	0.45138 (17)	0.0654 (5)	
C2	0.5223 (2)	0.26001 (14)	0.5541 (2)	0.0797 (6)	
H2	0.5810	0.2208	0.5936	0.096*	
C3	0.5326 (2)	0.33854 (15)	0.5978 (2)	0.0904 (7)	
H3	0.5980	0.3523	0.6682	0.108*	
C4	0.4468 (3)	0.39682 (15)	0.5381 (3)	0.0909 (7)	
H4	0.4543	0.4499	0.5677	0.109*	
C5	0.3502 (2)	0.37608 (16)	0.4348 (3)	0.0923 (7)	
H5	0.2922	0.4154	0.3942	0.111*	
C6	0.3380 (2)	0.29824 (14)	0.3907 (2)	0.0764 (6)	
H6	0.2723	0.2845	0.3205	0.092*	
C7	0.49373 (17)	0.10245 (12)	0.39629 (16)	0.0616 (5)	
C8	0.42311 (18)	0.03487 (12)	0.34748 (17)	0.0659 (5)	
C9	0.4821 (2)	−0.03604 (13)	0.31905 (17)	0.0681 (5)	
C10	0.67331 (18)	0.04538 (13)	0.39924 (17)	0.0670 (5)	
C11	0.6857 (6)	−0.0970 (5)	0.3246 (5)	0.0795 (18)	0.77
H11A	0.6320	−0.1448	0.3109	0.095*	0.77
H11B	0.7589	−0.1073	0.3956	0.095*	0.77
C12	0.7294 (5)	−0.0808 (4)	0.2082 (5)	0.138 (2)	0.77
H12A	0.7829	−0.0328	0.2238	0.166*	0.77
H12B	0.7810	−0.1263	0.1980	0.166*	0.77
C13	0.6379 (6)	−0.0699 (3)	0.1027 (5)	0.149 (2)	0.77
H13A	0.6006	−0.0168	0.1050	0.179*	0.77
H13B	0.5726	−0.1101	0.0979	0.179*	0.77
C14	0.6776 (10)	−0.0757 (5)	−0.0175 (6)	0.142 (4)	0.77
H14A	0.7472	−0.0395	−0.0114	0.213*	0.77
H14B	0.6074	−0.0606	−0.0874	0.213*	0.77
H14C	0.7026	−0.1304	−0.0279	0.213*	0.77
C11'	0.704 (2)	−0.0825 (18)	0.3141 (15)	0.104 (10)	0.23
H11C	0.7847	−0.0555	0.3291	0.125*	0.23
H11D	0.7168	−0.1296	0.3679	0.125*	0.23
C12'	0.6617 (12)	−0.1107 (8)	0.1849 (10)	0.081 (3)	0.23
H12C	0.6172	−0.1610	0.1880	0.097*	0.23
H12D	0.5967	−0.0720	0.1431	0.097*	0.23
C13'	0.7276 (11)	−0.1267 (7)	0.0973 (8)	0.104 (4)	0.23
H13C	0.8104	−0.1024	0.1329	0.125*	0.23
H13D	0.7414	−0.1850	0.1015	0.125*	0.23
C14'	0.695 (2)	−0.1081 (15)	−0.0281 (18)	0.101 (6)	0.23
H14D	0.6086	−0.1234	−0.0676	0.152*	0.23
H14E	0.7492	−0.1374	−0.0650	0.152*	0.23
H14F	0.7046	−0.0508	−0.0381	0.152*	0.23
C15	0.87510 (18)	0.10857 (13)	0.4721 (2)	0.0717 (6)	
C16	0.9372 (2)	0.14531 (16)	0.4005 (2)	0.0856 (7)	
H16	0.9238	0.1289	0.3191	0.103*	
C17	1.0212 (2)	0.20782 (16)	0.4509 (2)	0.0895 (7)	
H17	1.0632	0.2338	0.4020	0.107*	
C18	1.04316 (19)	0.23192 (14)	0.5710 (2)	0.0809 (6)	
C19	0.9782 (2)	0.19289 (17)	0.6399 (2)	0.0904 (7)	
H19	0.9913	0.2088	0.7215	0.108*	
C20	0.8938 (2)	0.13042 (16)	0.5914 (2)	0.0865 (7)	
H20	0.8511	0.1042	0.6396	0.104*	
C21	1.1367 (3)	0.29832 (18)	0.6258 (3)	0.1124 (9)	
H21A	1.2173	0.2847	0.6163	0.169*	
H21B	1.1449	0.3040	0.7121	0.169*	
H21C	1.1074	0.3486	0.5840	0.169*	
N1	0.40888 (13)	0.15838 (10)	0.40749 (14)	0.0651 (4)	
N2	0.28911 (15)	0.12506 (12)	0.36653 (17)	0.0760 (5)	
N3	0.29856 (15)	0.05057 (12)	0.33090 (16)	0.0763 (5)	
N4	0.61476 (15)	−0.02544 (10)	0.34970 (14)	0.0686 (5)	
N5	0.62063 (14)	0.11114 (10)	0.42446 (15)	0.0660 (4)	
O1	0.43198 (15)	−0.09929 (10)	0.27448 (15)	0.0877 (5)	
O2	0.79833 (13)	0.04150 (9)	0.41997 (15)	0.0840 (5)	

### Atomic displacement parameters (Å^2^)

**Table:**

	*U*^11^	*U*^22^	*U*^33^	*U*^12^	*U*^13^	*U*^23^
C1	0.0492 (10)	0.0861 (14)	0.0599 (11)	0.0031 (9)	0.0152 (8)	0.0080 (10)
C2	0.0634 (12)	0.0853 (15)	0.0760 (14)	0.0001 (11)	−0.0002 (10)	0.0097 (11)
C3	0.0771 (15)	0.0954 (17)	0.0843 (15)	−0.0114 (13)	0.0029 (12)	0.0014 (13)
C4	0.0866 (17)	0.0855 (15)	0.1014 (19)	−0.0009 (13)	0.0299 (14)	0.0018 (13)
C5	0.0803 (16)	0.0996 (18)	0.0938 (18)	0.0185 (13)	0.0215 (13)	0.0128 (14)
C6	0.0601 (12)	0.0930 (16)	0.0707 (13)	0.0125 (10)	0.0115 (10)	0.0046 (11)
C7	0.0500 (10)	0.0810 (12)	0.0489 (10)	−0.0003 (9)	0.0079 (7)	0.0085 (8)
C8	0.0558 (11)	0.0851 (13)	0.0507 (10)	−0.0027 (9)	0.0068 (8)	0.0085 (9)
C9	0.0658 (12)	0.0824 (14)	0.0495 (10)	−0.0053 (10)	0.0074 (8)	0.0065 (9)
C10	0.0532 (11)	0.0879 (14)	0.0570 (11)	0.0019 (10)	0.0121 (8)	0.0019 (10)
C11	0.075 (2)	0.086 (3)	0.076 (3)	0.011 (3)	0.020 (2)	−0.004 (2)
C12	0.111 (4)	0.156 (5)	0.147 (5)	−0.007 (3)	0.038 (4)	−0.070 (4)
C13	0.205 (6)	0.147 (4)	0.114 (3)	0.064 (4)	0.077 (4)	0.047 (3)
C14	0.200 (8)	0.147 (7)	0.095 (4)	−0.014 (5)	0.069 (4)	0.014 (4)
C11'	0.129 (17)	0.097 (15)	0.105 (15)	−0.015 (10)	0.064 (12)	−0.019 (10)
C12'	0.077 (7)	0.097 (8)	0.069 (7)	−0.011 (6)	0.022 (6)	−0.018 (6)
C13'	0.115 (10)	0.090 (7)	0.097 (9)	0.002 (7)	0.015 (7)	−0.010 (6)
C14'	0.090 (10)	0.108 (15)	0.106 (12)	−0.014 (9)	0.030 (8)	0.001 (9)
C15	0.0446 (10)	0.0929 (14)	0.0758 (13)	0.0081 (10)	0.0154 (9)	−0.0068 (11)
C16	0.0669 (13)	0.1198 (18)	0.0734 (14)	0.0020 (13)	0.0264 (11)	−0.0123 (13)
C17	0.0689 (14)	0.1146 (18)	0.0916 (17)	−0.0009 (13)	0.0341 (12)	−0.0005 (14)
C18	0.0544 (11)	0.0949 (15)	0.0930 (16)	0.0076 (11)	0.0217 (11)	−0.0096 (12)
C19	0.0702 (14)	0.127 (2)	0.0742 (14)	−0.0021 (13)	0.0221 (11)	−0.0188 (13)
C20	0.0644 (13)	0.1234 (19)	0.0741 (15)	−0.0102 (13)	0.0247 (11)	−0.0068 (13)
C21	0.0855 (18)	0.119 (2)	0.133 (2)	−0.0136 (16)	0.0334 (17)	−0.0260 (18)
N1	0.0459 (8)	0.0832 (11)	0.0604 (9)	0.0038 (8)	0.0072 (7)	0.0055 (8)
N2	0.0471 (9)	0.0998 (13)	0.0749 (11)	−0.0033 (8)	0.0091 (8)	0.0027 (9)
N3	0.0534 (10)	0.0983 (13)	0.0695 (10)	−0.0053 (9)	0.0072 (8)	0.0046 (9)
N4	0.0641 (10)	0.0817 (11)	0.0557 (9)	0.0031 (8)	0.0118 (7)	0.0001 (8)
N5	0.0480 (9)	0.0836 (11)	0.0630 (9)	0.0007 (8)	0.0116 (7)	0.0018 (8)
O1	0.0859 (11)	0.0879 (11)	0.0802 (10)	−0.0134 (8)	0.0117 (8)	−0.0049 (8)
O2	0.0526 (8)	0.1000 (11)	0.0981 (11)	0.0020 (7)	0.0209 (7)	−0.0187 (8)

### Geometric parameters (Å, °)

**Table:**

C1—C2	1.370 (3)	C14—H14B	0.9600
C1—C6	1.375 (3)	C14—H14C	0.9600
C1—N1	1.432 (3)	C11'—C12'	1.473 (10)
C2—C3	1.376 (3)	C11'—N4	1.502 (10)
C2—H2	0.9300	C11'—H11C	0.9700
C3—C4	1.376 (3)	C11'—H11D	0.9700
C3—H3	0.9300	C12'—C13'	1.426 (9)
C4—C5	1.371 (4)	C12'—H12C	0.9700
C4—H4	0.9300	C12'—H12D	0.9700
C5—C6	1.366 (3)	C13'—C14'	1.39 (2)
C5—H5	0.9300	C13'—H13C	0.9700
C6—H6	0.9300	C13'—H13D	0.9700
C7—N1	1.349 (2)	C14'—H14D	0.9600
C7—N5	1.354 (2)	C14'—H14E	0.9600
C7—C8	1.376 (3)	C14'—H14F	0.9600
C8—N3	1.362 (3)	C15—C20	1.355 (3)
C8—C9	1.422 (3)	C15—C16	1.356 (3)
C9—O1	1.215 (2)	C15—O2	1.410 (3)
C9—N4	1.416 (3)	C16—C17	1.389 (3)
C10—N5	1.302 (3)	C16—H16	0.9300
C10—O2	1.336 (2)	C17—C18	1.369 (3)
C10—N4	1.369 (3)	C17—H17	0.9300
C11—N4	1.491 (4)	C18—C19	1.373 (3)
C11—C12	1.562 (8)	C18—C21	1.505 (3)
C11—H11A	0.9700	C19—C20	1.387 (3)
C11—H11B	0.9700	C19—H19	0.9300
C12—C13	1.326 (6)	C20—H20	0.9300
C12—H12A	0.9700	C21—H21A	0.9600
C12—H12B	0.9700	C21—H21B	0.9600
C13—C14	1.559 (7)	C21—H21C	0.9600
C13—H13A	0.9700	N1—N2	1.381 (2)
C13—H13B	0.9700	N2—N3	1.304 (3)
C14—H14A	0.9600		
			
C2—C1—C6	121.1 (2)	C13'—C12'—H12C	104.1
C2—C1—N1	119.77 (18)	C11'—C12'—H12C	104.1
C6—C1—N1	119.07 (18)	C13'—C12'—H12D	104.1
C1—C2—C3	118.9 (2)	C11'—C12'—H12D	104.1
C1—C2—H2	120.5	H12C—C12'—H12D	105.5
C3—C2—H2	120.5	C14'—C13'—C12'	130.0 (14)
C4—C3—C2	120.5 (2)	C14'—C13'—H13C	104.8
C4—C3—H3	119.8	C12'—C13'—H13C	104.8
C2—C3—H3	119.8	C14'—C13'—H13D	104.8
C5—C4—C3	119.5 (2)	C12'—C13'—H13D	104.8
C5—C4—H4	120.2	H13C—C13'—H13D	105.8
C3—C4—H4	120.2	C13'—C14'—H14D	109.5
C6—C5—C4	120.8 (2)	C13'—C14'—H14E	109.5
C6—C5—H5	119.6	H14D—C14'—H14E	109.5
C4—C5—H5	119.6	C13'—C14'—H14F	109.5
C5—C6—C1	119.1 (2)	H14D—C14'—H14F	109.5
C5—C6—H6	120.4	H14E—C14'—H14F	109.5
C1—C6—H6	120.4	C20—C15—C16	121.9 (2)
N1—C7—N5	127.68 (18)	C20—C15—O2	121.1 (2)
N1—C7—C8	105.08 (17)	C16—C15—O2	116.70 (19)
N5—C7—C8	127.23 (19)	C15—C16—C17	118.9 (2)
N3—C8—C7	109.43 (18)	C15—C16—H16	120.6
N3—C8—C9	129.96 (19)	C17—C16—H16	120.6
C7—C8—C9	120.58 (19)	C18—C17—C16	121.3 (2)
O1—C9—N4	121.2 (2)	C18—C17—H17	119.4
O1—C9—C8	127.7 (2)	C16—C17—H17	119.4
N4—C9—C8	111.04 (18)	C17—C18—C19	117.8 (2)
N5—C10—O2	120.81 (18)	C17—C18—C21	120.9 (2)
N5—C10—N4	127.48 (18)	C19—C18—C21	121.3 (2)
O2—C10—N4	111.70 (18)	C18—C19—C20	121.9 (2)
N4—C11—C12	110.1 (5)	C18—C19—H19	119.0
N4—C11—H11A	109.6	C20—C19—H19	119.0
C12—C11—H11A	109.6	C15—C20—C19	118.2 (2)
N4—C11—H11B	109.6	C15—C20—H20	120.9
C12—C11—H11B	109.6	C19—C20—H20	120.9
H11A—C11—H11B	108.2	C18—C21—H21A	109.5
C13—C12—C11	115.9 (5)	C18—C21—H21B	109.5
C13—C12—H12A	108.3	H21A—C21—H21B	109.5
C11—C12—H12A	108.3	C18—C21—H21C	109.5
C13—C12—H12B	108.3	H21A—C21—H21C	109.5
C11—C12—H12B	108.3	H21B—C21—H21C	109.5
H12A—C12—H12B	107.4	C7—N1—N2	108.99 (17)
C12—C13—C14	116.1 (6)	C7—N1—C1	131.55 (16)
C12—C13—H13A	108.3	N2—N1—C1	119.47 (15)
C14—C13—H13A	108.3	N3—N2—N1	108.51 (16)
C12—C13—H13B	108.3	N2—N3—C8	107.99 (16)
C14—C13—H13B	108.3	C10—N4—C9	122.28 (17)
H13A—C13—H13B	107.4	C10—N4—C11	122.6 (4)
C12'—C11'—N4	115.4 (11)	C9—N4—C11	115.2 (4)
C12'—C11'—H11C	108.4	C10—N4—C11'	111.9 (13)
N4—C11'—H11C	108.4	C9—N4—C11'	125.1 (12)
C12'—C11'—H11D	108.4	C11—N4—C11'	13.6 (18)
N4—C11'—H11D	108.4	C10—N5—C7	111.38 (17)
H11C—C11'—H11D	107.5	C10—O2—C15	119.97 (16)
C13'—C12'—C11'	132.7 (14)		

### Hydrogen-bond geometry (Å, °)

**Table:**

*D*—H···*A*	*D*—H	H···*A*	*D*···*A*	*D*—H···*A*
C12—H12A···O2	0.97	2.50	3.048 (5)	116
C2—H2···O1^i^	0.93	2.53	3.230 (3)	133
C3—H3···N2^ii^	0.93	2.61	3.535 (2)	174

Symmetry codes: (i) −*x*+1, −*y*, −*z*+1; (ii) *x*+1/2, −*y*+1/2, *z*+1/2.
